# Retraction: Biogenic sunflower oil-chitosan decorated fly ash nanocomposite film using white shrimp shell waste: Antibacterial and immunomodulatory potential

**DOI:** 10.1371/journal.pone.0351919

**Published:** 2026-06-23

**Authors:** The PLOS One Editors

After this article [1,2] was published, concerns were raised regarding results presented in Figs 4, 9, 11, and 12.

Specifically:

- The following panels appear to fully or partially overlap:
  - Fig 4b and Fig 4d
  - Figs 9d and 9e in [1, 2] and Fig 5A in [3]
  - Fig 11d p-JNK in [1, 2] and Fig 4 p-JNK left panel in [4]*
  - Fig 11d JNK in [1, 2] and Fig 4 JNK left panel in [4]*
  - Fig 11d GAPDH in [1, 2] and Fig 4 GAPDH left panel in [4]*
  - Fig 11e p-ERK1/2 in [1, 2] and Fig 3A p-ERK1/2 panel in [5]*
  - Fig 11e ERK1/2 in [1, 2] and Fig 5A t-ERK1/2 in [6]
  - Fig 11e GAPDH in [1, 2] and Fig 5B GAPDH in [6]
- There appear to be repetitive areas within one of more panels in the following figures in [1]:
  - Fig 9
  - Fig 11
  - Fig 12

Corresponding author MA stated they disagreed with the above concerns about repetitive areas within Figs 9, 11, and 12, and the concerns for Figs 4b and 4d. They provided incomplete underlying data for Figs 4, 9, 11, and 12. Upon editorial review, the comments and data provided by the authors in response to the above-mentioned issues did not resolve the concerns about the published results.

In light of the above concerns that question the reliability and integrity of the reported results, the *PLOS One* Editors retract this article.

MA did not agree with the retraction. SSA and MFET either did not respond directly or could not be reached.

The Fig 11d p-JNK, JNK, and GAPDH and Fig 11e p-ERK1/2 panels appear similar to previously published materials by different authors that were not offered under a CC BY license, and are therefore excluded from this article’s [1] license. The * by the citations above marks the oldest publication of each respective image of which PLOS is aware.
